# Development of the excitation-contraction coupling machinery and its relation to myofibrillogenesis in human iPSC-derived skeletal myocytes

**DOI:** 10.1186/s13395-017-0147-5

**Published:** 2018-01-05

**Authors:** Jeanne Lainé, Gunnar Skoglund, Emmanuel Fournier, Nacira Tabti

**Affiliations:** 1 Département de Physiologie, Faculté de Médecine Pierre & Marie Curie site Pitié-Salpêtrière, UPMC, 91, Bd de l’Hôpital, 75013 Paris, France; 2 UPEC, Créteil, France

**Keywords:** iPS-derived skeletal muscle cells, Excitation-contraction coupling, Human-induced pluripotent stem cells, RyR1, Cav1.1, Ca^2+^ signaling, SR-TT junctions, Myofibrillogenesis

## Abstract

**Background:**

Human induced pluripotent stem cells-derived myogenic progenitors develop functional and ultrastructural features typical of skeletal muscle when differentiated in culture. Besides disease-modeling, such a system can be used to clarify basic aspects of human skeletal muscle development. In the present study, we focus on the development of the excitation-contraction (E-C) coupling, a process that is essential both in muscle physiology and as a tool to differentiate between the skeletal and cardiac muscle. The occurrence and maturation of E-C coupling structures (Sarcoplasmic Reticulum-Transverse Tubule (SR-TT) junctions), key molecular components, and Ca^2+^ signaling were examined, along with myofibrillogenesis.

**Methods:**

Pax7^+^-myogenic progenitors were differentiated in culture, and developmental changes were examined from a few days up to several weeks. Ion channels directly involved in the skeletal muscle E-C coupling (RyR1 and Cav1.1 voltage-gated Ca^2+^ channels) were labeled using indirect immunofluorescence. Ultrastructural changes of differentiating cells were visualized by transmission electron microscopy. On the functional side, depolarization-induced intracellular Ca^2+^ transients mediating E-C coupling were recorded using Fura-2 ratiometric Ca^2+^ imaging, and myocyte contraction was captured by digital photomicrography.

**Results:**

We show that the E-C coupling machinery occurs and operates within a few days post-differentiation, as soon as the myofilaments align. However, Ca^2+^ transients become effective in triggering myocyte contraction after 1 week of differentiation, when nascent myofibrils show alternate A-I bands. At later stages, myofibrils become fully organized into adult-like sarcomeres but SR-TT junctions do not reach their triadic structure and typical A-I location. This is mirrored by the absence of cross-striated distribution pattern of both RyR1 and Cav1.1 channels.

**Conclusions:**

The E-C coupling machinery occurs and operates within the first week of muscle cells differentiation. However, while early development of SR-TT junctions is coordinated with that of nascent myofibrils, their respective maturation is not. Formation of typical triads requires other factors/conditions, and this should be taken into account when using in-vitro models to explore skeletal muscle diseases, especially those affecting E-C coupling.

**Electronic supplementary material:**

The online version of this article (10.1186/s13395-017-0147-5) contains supplementary material, which is available to authorized users.

## Background

Excitation-contraction coupling is a central process in the muscle which translates plasma membrane electrical signals into a rise in intracellular Ca^2+^ that triggers myofilament sliding and muscle contraction [1–3].

During skeletal muscle development, the occurrence of this process depends on the formation of specific junctions between the sarcoplasmic reticulum (SR) and the plasma membrane or its invaginations, the T-tubules (TT) [4, 5]. Communication between apposed plasma and SR membranes implies expression and close interaction between the voltage-gated L-type Ca^2+^ channel type 1 (Cav1.1) also known as α_1_s-dihydropyridine receptor (α_1_s-DHPR) and the ryanodine receptor type 1 (RyR1), respectively [6–8]. A depolarization-triggered conformational change of Cav1.1 during sarcolemma excitation brings about the opening of RyR1 channels and the release of Ca^2+^ ions from the SR lumen to the cytosol [9–11].

We have recently shown that myogenic progenitors derived from human induced pluripotent stem cells (iPSC) acquire specific skeletal muscle E-C coupling within the first 2 weeks of their differentiation in vitro [12]. Intracellular Ca^2+^ transients could be elicited by plasma membrane depolarization in the absence of extracellular Ca^2+^, which represents a landmark of E-C signaling in the skeletal as opposed to the cardiac muscle [2, 13]. This was corroborated by ultrastructural analysis showing typical skeletal muscle sarcomeric organization and the presence of SR-TT junctions with unequivocal rows of RyR feet.

Human iPSC-derived skeletal muscle cells offer new opportunities for clarifying basic aspects of skeletal muscle development in humans, which is by far the least documented mammalian species. Such knowledge can in turn be used to appraise and dissect pathogenic processes in the emerging iPSC-based skeletal muscle models [12, 14–17]. The present study focuses on the development of the E-C coupling machinery and its relation to myofibrillogenesis, with the purpose of answering the following questions:

(1) When do the structures supporting E-C coupling, i.e., SR-TT junctions, occur, and how do they develop? How does this correlate with myofibrillogenesis?; (2) When do key ion channels involved in skeletal muscle E-C coupling (Cav1.1 and RyR1) appear after switching to differentiation conditions? What is their subcellular distribution, and does it change with skeletal myocyte maturation?; and (3) When does the voltage-induced intracellular Ca^2+^ transient that mediates EC coupling occur? How does it develop, and when does it generate effective cell contraction?

To answer these questions, we have performed conventional immunofluorescence and electron microscopy experiments on differentiating human iPSC-derived skeletal myocytes in order to detect the first signs of E-C coupling machinery and myofilament assembly and follow their respective changes over time. In parallel, we recorded depolarization-induced intracellular Ca^2+^ transients to correlate the development of E-C coupling structures and components with their signaling function.

## Methods

### Culture of iPSC-derived skeletal myocytes

Myogenic progenitors were developed in R. Perlingeiro’s laboratory [15]. Briefly, A fully characterized human iPS cell line was stably transfected with an inducible lentiviral Pax7 (iPax7) to produce a large number of myogenic cells. The lentiviral construct contains a tet-responsive element (TRE) to induce Pax7 expression by doxycycline (dox) and an IRES-EGFP reporter to detect transgene expression through green fluorescence. Pax7^+^GFP^+^ cells were expanded in proliferation medium consisting of IMDM-glutamax supplemented with 15% fetal bovine serum, 10% horse serum, 1% penicillin-streptomycin, 5 ng/ml BFGF (Gibco), 50 μg/ml ascorbic acid, 4.5 mM monothioglycerol, and 0.75 μg/ml Dox (SIGMA). Myogenic progenitors were plated on glass or plastic (Themanox, Nunc) coverslips coated overnight with 0.1% gelatin (SIGMA) and 2.4 μg/ml laminin (GIBCO). The latter was newly introduced, which resulted in a better alignment of the myocytes and a slight hastening of their development as compared to gelatin alone. When tight confluence was reached, cells were differentiated by switching to a low glucose DMEM (Gibco) containing 2% horse serum, 1% penicillin-streptomycin, and 1% insulin-transferrin-selenium (Gibco). Media were renewed twice a week. Further details on the cell culture procedure can be found in our previous report [12].

### Immunofluorescence labeling

Cells were fixed at room temperature in 3% paraformaldehyde and 4% sucrose, and subsequently permeabilized (0.5% Triton X100 in PBS) for 10 min, and incubated in the blocking solution (5% bovine serum albumin and 0.1% Triton X100 in PBS) for 30 min. Between each step, cells were rinsed with PBS containing 0.1% Triton X100 for 10 min. Primary mouse antibodies were applied overnight at 4 °C and diluted (*v*/*v*) as follows: α-actinin (1/500, SIGMA clone EA-53), RyR1 (1/100, Thermofisher clone 34C), or Cav1.1 (1/100, Thermofisher, clone 1A). Alexa Fluor conjugated goat anti-mouse IgG antibodies (Invitrogen, 1/1000) were then applied in the blocking solution for 1 h at room temperature. At last, the nuclei were stained with Hoechst, and the coverslips mounted in a drop of Fluoromount-G (SouthernBiotech). Conventional fluorescence examination was performed through an oil immersion ×60 lens objective (NA 1.40) on TE 2000-S Eclipse Nikon microscope, and images were acquired using DS-Qi1 cooled digital camera and NIS software (Nikon). The size of the fluorescent particles was measured using ImageJ software (NIH, USA).

### Transmission electron microscopy

For electron microscopy (EM) examination, cells were cultured on Thermanox coverslips (Nunc, Rochester, USA). Fixation medium contained 0.1 M phosphate buffer containing 2% glutaraldehyde and 2% paraformaldehyde (pH 7.4) and was applied for 30 min at room temperature. Fixed cells were subsequently treated with 2% OsO4 in phosphate buffer, gradually dehydrated in acetone with a staining step in 1% uranyl acetate and 70% acetone. Cells were embedded in Epon resin (EMS, Fort Washington, PA, USA), and the block was subsequently cut into ultrathin sections. The latter were collected on grids and stained with uranyl acetate and lead citrate. Cells were examined with Philips CM120 electron microscope (Philips, Eindhoven, Netherlands) operated at 80 kV and photographed with SIS Morada digital camera (Olympus, Münster, Germany); see reference [12] for further details.

### Intracellular Ca^2+^ measurements

Intracellular calcium signals were induced by membrane depolarization with high extracellular K^+^ and measured using fura-2-based microfluorometry [18]. Cells used for Ca^2+^ measurements were cultured and differentiated in the same way as those used for immunofluorescence labeling (see above). Prior to Ca^2+^ measurements, cells were loaded with 4 μM fura-2/AM and 0.01% pluronic acid (Molecular Probes, Eugene, OR) for 30–45 min at room temperature in standard buffer containing 125 mM NaCl, 5 mM KCl, 1 mM MgCl2, 2 mM CaCl_2_, 10 mM HEPES, and 10 mM glucose (pH 7.4). Coverslips were placed in a specifically designed, thermostatically controlled (37 °C) perfusion chamber mounted onto a Nikon inverted epifluorescence microscope. Cells were viewed with a 40.3/1.3 NA glycerol immersion Zeiss objective or an oil immersion Nikon objective (40.3/1.3 NA). Signals were recorded using a video imaging system (AquaCosmos, Hamamatsu Photonics) for dual wavelength excitation fluorometry (alternating excitation 340/380 nm; emission 510 nm). Images were acquired at 1.2–1.6 s time intervals for up to 15 min. Cells loaded with fura-2 were first perfused with Ca^2+^-free/normal extracellular K^+^ medium for 4 min to clear out Ca^2+^ ions from the extracellular space and enable baseline stabilization. Membrane depolarization was induced by perfusing the cells with an extracellular medium containing 50 mM K^+^ for 4 min to enable full rise and decay of the Ca^2+^ transient. Changes in [Ca^2+^]_i_ were assessed by the fluorescence ratio R_340/380_, which directly reflects the changes in free intracellular Ca^2+^ [18]; see [12] for further details. All cells within a field were recorded, and signals were analyzed from all cells responding to membrane depolarization.

The Ca^2+^ signal obtained from individual cells was normalized to the baseline mean value and expressed as a percentage ((Signal R_340/380_/mean baseline R_340/380)_) × 100). The baseline mean value was obtained by averaging all the data points over 20 s prior to membrane depolarization.

The signal obtained for each individual cell was integrated over the first 30 s of membrane depolarization using Origin Pro software (OriginLab Corporation). This provided the integrated fluorescence (area under the curve), which reflects the total amount of Ca^2+^ released along with two other characteristics: the maximum fluorescence ratio relative to the baseline (maximum stimulation), which indicates the highest level of [Ca^2+^] attained during membrane depolarization, and the signal time to peak, which reflects the rate of Ca^2+^ release from the SR. Notice that the mean values depicted in Fig. 5B–Dhistograms may show some slight differences with those that can be inferred from the averaged signals in Fig. 5A. This is explained by the different methods used to process the data; In histograms of Fig. 5B–D, the mean values were obtained by processing each individual signal and then averaging the values of each characteristic, whereas in Fig. 5A, the signals represent averaged data at specific time points. Nevertheless, this had no impact on the statistical analysis. Exponential fittings of the Ca^2+^ signal decay phase were made after normalization of the signal to enable comparison between different datasets.

### Statistical analysis

At least three independent experiments were carried out for each time point regardless of the method used (immunofluorescence, electron microscopy, or Ca^2+^ imaging). Quantitative data are given as mean ± SEM. Unpaired, two-tailed Student’s *t* test was used for comparing two groups. One-way ANOVA was used to detect differences between intracellular Ca^2+^ signals obtained at different stages of myocytes differentiation. A post hoc Tukey’s test was subsequently used to compare data obtained at each differentiation time point with those obtained at late stages (> 3 weeks). Analyses were made using STATISTICA software (StatSoft Inc.).

## Results

To describe the onset and development of the EC-coupling machinery and of the myofibrils during the differentiation of human iPSC-derived myogenic progenitors into the skeletal muscle cells, we have explored three successive periods: an early stage (from day 3 through day 5) when rapid changes occur, an intermediate time point (day 7) when most of the skeletal muscle landmarks are settled, and a late period that extends from 3 to 8 weeks post-differentiation, during which skeletal myocytes have reached steady maturation with no variation in their differentiation state at least regarding the features investigated herein. These cells will be referred to as mature cells.

### Development of myofibrils and E-C coupling junctions

**Fig. 1:**
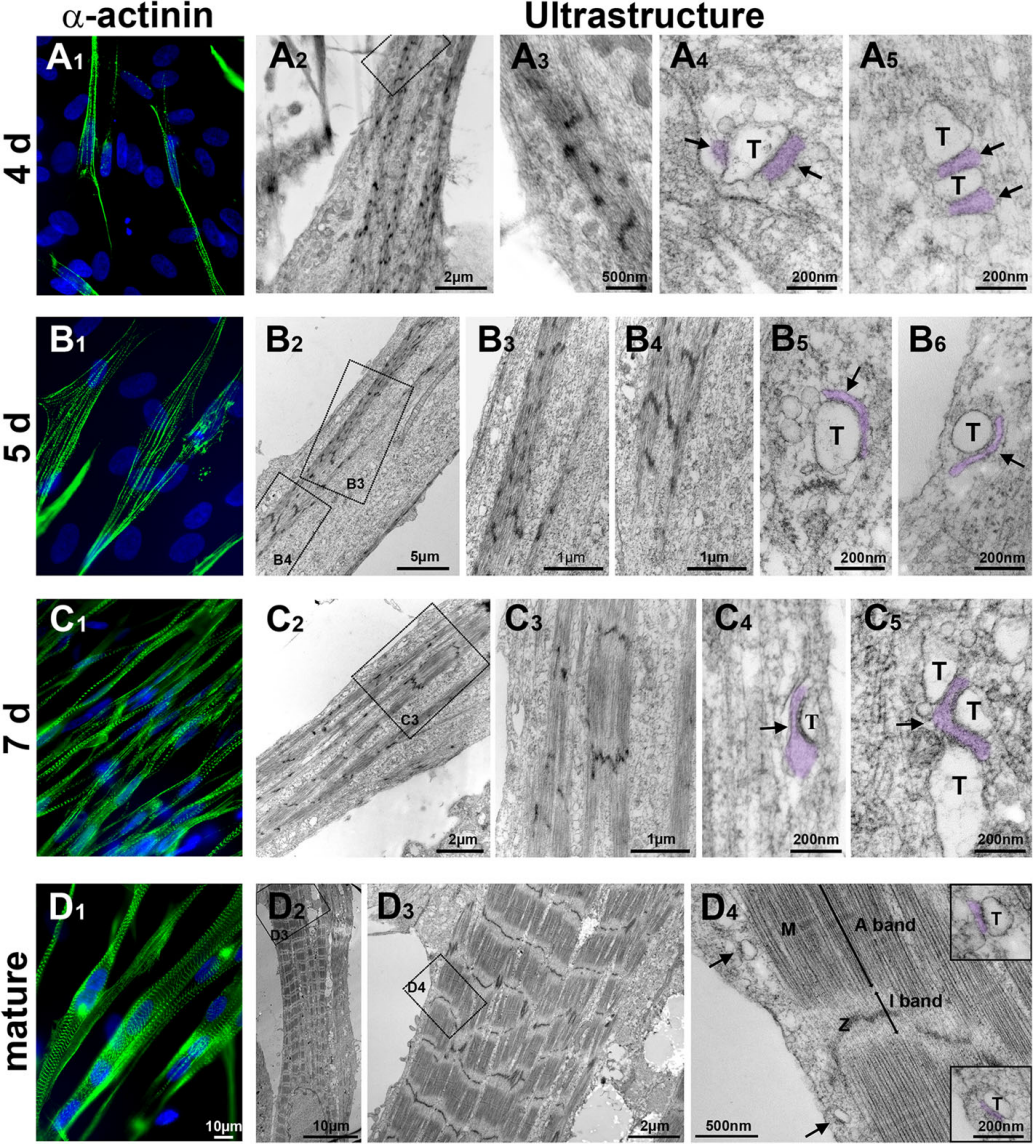
Myofibrillogenesis and SR-TT junctions in differentiating human iPSC-derived skeletal myocytes. **A1**–**A5** Day 4 post-differentiation. Immunofluorescence labeling of α-actinin (**A1**) and electron micrographs of ultrathin longitudinal cell sections (**A2**–**A5**). Nascent myofibrils with Z-bodies (**A2**–**A3**); the boxed area is enlarged in A3 for further details. **A4**–**A5** SR-TT junctions from different cells. Whenever possible, the SR is highlighted by artificial post-coloring. Note that T-tubules (T) are surrounded and/or associated with numerous caveolae. **B1**–**B6** Day 5 post-differentiation. Transition from Z-bodies to Z-bands as revealed by α-actinin labeling (**B1**) and confirmed by electron microscopy (**B2**–**B4**). Upper and lower boxed areas in **B2** are enlarged in **B3** and **B4**, as indicated. **B5**–**B6** SR-TT junctions from different cells; feet-like structures are visible and caveolae surround the T-tubules. **C1**–**C5** Day 7 post-differentiation. A significant increase in the number of α-actinin+ cells with prevalent Z-bands (**C1**). **C2**–**C3** Myofibrils with well-defined A-I banding pattern; the boxed area is enlarged in **C3**. **C4**–**C5** Large and multiple SR-TT junctions from different cells with noticeable rows of feet-like structures in the interspace. **D1**–**D4** Mature stages (> 3 weeks post-differentiation). **D1** Alpha-actinin labeling at day 26 post-differentiation showing large skeletal myocytes with clear-cut Z-lines. **D2**–**D3** Ultrastructure of 29-day-old myocytes showing parallel bundles of myofibrils (**D2**) with fully mature and aligned banding pattern including H-bands and M-lines as can be seen from subsequent enlargement of the boxed areas in **D3** and **D4**. SR-TT junctions (arrows in **D4**) are enlarged in boxes on the right. Notice the smaller size of these structures and their proximity to the myofibrils compared with earlier stages

Changes in cell morphology during differentiation were first noticed by phase contrast microscopy in routine cell culture observation (Additional file 1: Figure S1). To readily detect any cytoskeleton rearrangement towards a sarcomeric pattern, immunofluorescence labeling of α-actinin was carried out at different times of cell differentiation. This was refined by ultrastructural analysis of the cells, which enabled a detailed assessment of myofibrillogenesis as well as the identification of nascent E-C coupling structures (SR-TT junctions) and their change with development (Fig. 1, Additional file 2: Figure S2).

Human iPSC-derived myogenic progenitors divide rapidly (~one division/30 h) in the proliferation medium (high-serum media) and ultimately form a tight layer of flat cells with round prominent nucleus (Additional file 1: Figure S1A). Following the switch to the differentiation medium, cells start elongating and aligning in a preferential direction (Additional file 1: Figure S1). Immunolabeling with α-actinin together with Hoechst staining of the nuclei provided an easy way of counting the percentage of cells with cytoskeleton remodeling towards a myofibrillar pattern and to follow the changes with time of differentiation in culture (Fig. 1).

On day 3 post-differentiation, only ~ 12% of the cells (Fig. 4) showed labeling for α-actinin in the form of small puncta along their edge. Superimposition of fluorescent and bright field images indicated that α-actinin is expressed as soon as cells elongate (data not shown). At the ultrastructural level, most cells still displayed embryonic features (large nucleus, extended cisternae of rough endoplasmic reticulum), but a few displayed early myofilament assembly with bundles of mixed thick and thin filaments; no SR-TT junctions could be found (Additional file 2: Figure S2B).

On day 4, the number of elongated cells increased (Additional file 1: Figure S1), as did the proportion of cells showing α-actinin punctuate labeling (Fig. 1A_1_), which reached ~ 22% of the total cell population (Fig. 4). Ultrastructural examination revealed long bundles of mixed thick and thin filaments, which were mainly located at the periphery, close to the plasma membrane or along the nucleus (Fig. 1A_2,3_). Dense structures of irregular shape, presumably Z-bodies, appeared along these bundles and co-existed now and then with nascent Z-bands. Although recurrent, these structures did not delineate a regular sarcomeric pattern; A and I bands could not be clearly identified, and H-band and M-line were unseen. Nuclei had various shapes from oblate to clearly flattened. Appositions between SR and T-tubule-like structures were encountered at this stage (Fig. 1A_4,5_), but the junctional cleft was generally irregular and RyR feet could not be clearly discerned. On day 5 post-differentiation, α-actinin labeling was found in a higher proportion (~ 47%) of cells (Fig. 4) and appeared both as puncta and as short, irregular bands (Fig. 1B_1_). At the ultrastructural level, both Z-bodies and winding discontinuous Z-bands were found; however, there was generally no clear-cut A-I banding pattern apart from a few exceptions (Fig. 1B_2–4_). We also noticed that the most organized cytoskeleton pattern was preferentially located in the center, while nascent Z-bodies were scattered at the periphery of the cell (Additional file 3: Figure S3). This mixed pattern speaks in favor of a transition stage from myotube to myofiber. SR-TT junctions in the form of polyads, diads, or triads (often in the form of inverted triads: two SR cisternae and one T-tubule) were found in different areas of the cells and could be better delineated; RyR feet became recognizable in the gap that separates the two membranes (Fig. 1B_5,6_).

On day 7 post-differentiation, the large majority of the cells was elongated, had several long or oval nuclei predominantly at their edge, and their cross striations were readily visible in phase contrast microscopy (Additional file 1: Figure S1). Immunofluorescence labeling of α-actinin was readily visible and covered ~ 85% of the cells (Figs. 1C_1_ and 4 ). Nonetheless, close examination of the labeling revealed the presence of Z-bands that formed a zigzag pattern across the myofibrils rather than clear-cut Z-lines. These intermediate structures between early Z-bodies and mature Z-line were previously described in studies on myofibrillogenesis in chick embryos [19, 20]. Ultrastructural examination of 7-day-old myocytes showed that myofibrils were more frequently located at the center of the cells, some could still be found at the periphery, but their size was relatively smaller. Regardless of their location, myofibrils had a well-defined A-I banding pattern while the H-band could hardly be distinguished and the M-line remained undetectable (Fig. 1C_2,3_). SR-TT junctions with unambiguous rows of RyR feet were found in different areas of the cells (Fig. 1C_4,5_). These structures were relatively large and were composed of extended SR cisternae and T-tubules with a wide lumen, often surrounded and/or fused to caveolae. Diads, inverted triads as well as classical triads were encountered, but none followed an ordered distribution pattern in register with the myofibrils.

From day 14 post-differentiation, the proportion of α-actinin-labeled cells reached a steady level of about 80% (Fig. 4). Other cell types with embryonic features (flat shape, extended contours, and single prominent nucleus) were still present beneath the muscle cell layer, but their number remained stable throughout the maturation period. Such cells may correspond either to non-myogenic cell types (fibroblasts, iPSC) or to myogenic cells in a quiescent state. It is worth noting that the coexistence of non-myogenic cells could be beneficial for the differentiation of the predominant population of skeletal myocytes. Such idea is supported by a recent paper showing that myogenic progenitors interact with other cell types to regulate the extracellular matrix [21].

**Fig. 2:**
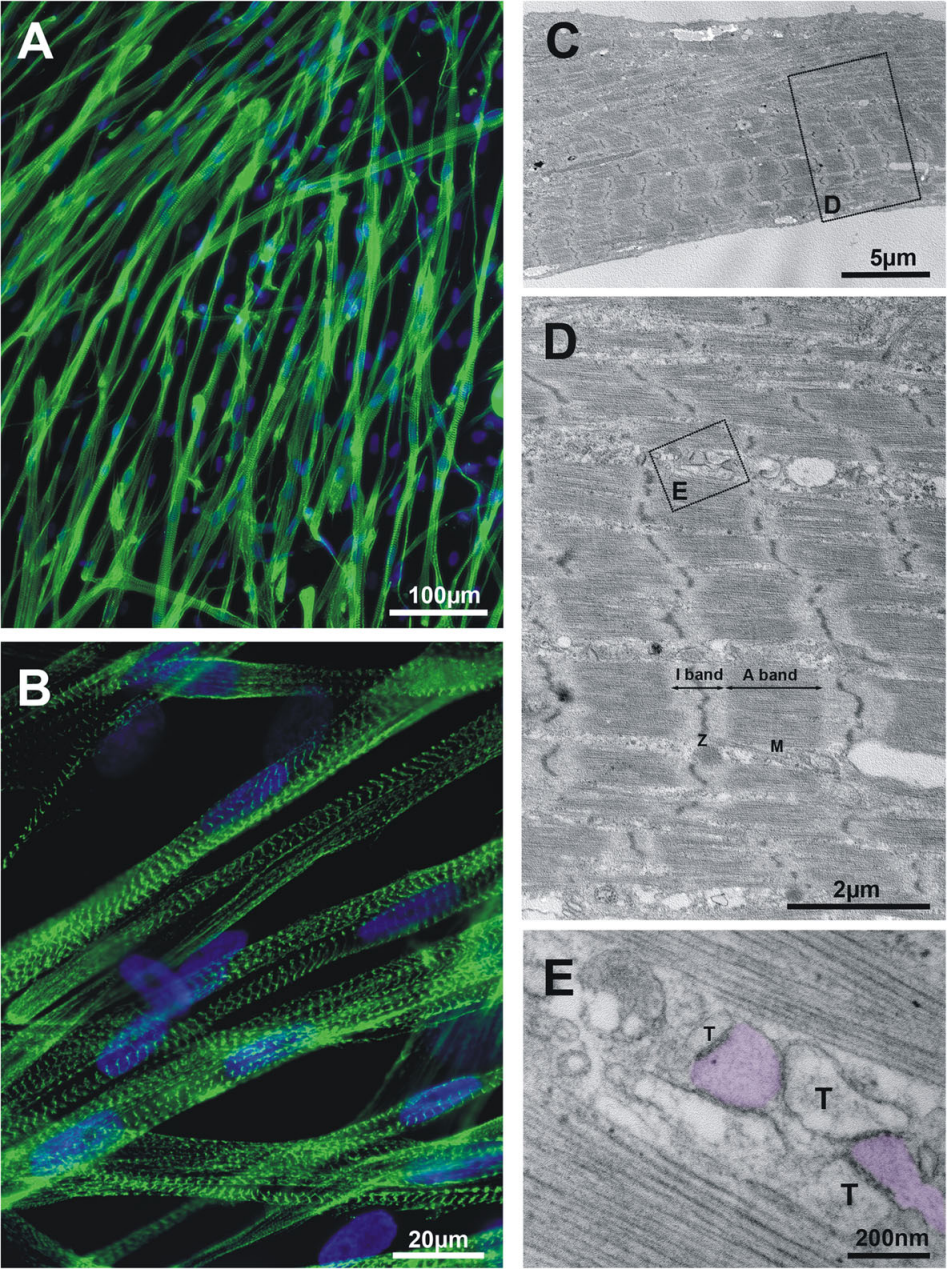
Alpha-actinin immunofluorescence and ultrastructure of human iPSC-derived skeletal myocytes on day 14 post-differentiation in culture. The left panel shows immunofluorescence labeling of α-actinin photographed at low (**A**) or high (**B**) magnification. The right panel depicts the ultrastructure of 14-day-old skeletal myocytes at increasing magnifications from **C** to **E**; the boxes delineate regions that are subsequently enlarged. A general view of the sarcomeric banding pattern is presented in **C**, followed by a detailed image of the different bands, as indicated. Myofibrils full maturity is attested by the presence of H-bands and M-lines. Multiple junctions between SR elements and T-tubules are illustrated in **E**; note the granular aspect of the SR lumen and the presence of regularly spaced (∼ 25 nm) electron-opaque feet-like structures between SR and T-tubules apposed membranes

At 14 days post-differentiation, cells with fully organized myofibrils were often encountered in electron micrographs; however, SR-TT junctions were still immature and did not follow the myofibrils cross-striation pattern (Fig. 2). To ensure that SR-TT junction reached their maximum degree of maturation under the present conditions, we extended the period of differentiation beyond 3 weeks. The results obtained at this late period are illustrated in Fig. 1D. Alpha-actinin immunofluorescence labeling of mature iPS-derived skeletal myocytes showed sarcomeric repeats delimited by clear-cut Z-lines. Analysis of the Z-Z intervals in 22-day-old myocytes confirmed their periodic distribution and yielded a mean value for the sarcomeric length of 2.21 ± 0.07 μm (95 Z-Z intervals from 11 muscle cells and 3 independent experiments). At the ultrastructural level, myofibrils were fully organized in A/I alternating bands (Fig. 1D_2,3_); H zones with central M-lines were readily detectable in the middle of the A-band (Fig. 1D_4_), similar to what is usually observed in adult skeletal muscle [22]. SR-TT junctions had a smaller size and a more uniform structure, and triads with a more central location were observed. Nevertheless, the presence of typical triads with a transverse location at the A-I junction was not encountered. Sporadically, individual triads could be observed at typical locations, in register with the A-I junction, as in the adult muscle, but this was probably a random rather than an orderly event.

### Onset and development of Cav1.1 and RyR1 channels

**Fig. 3:**
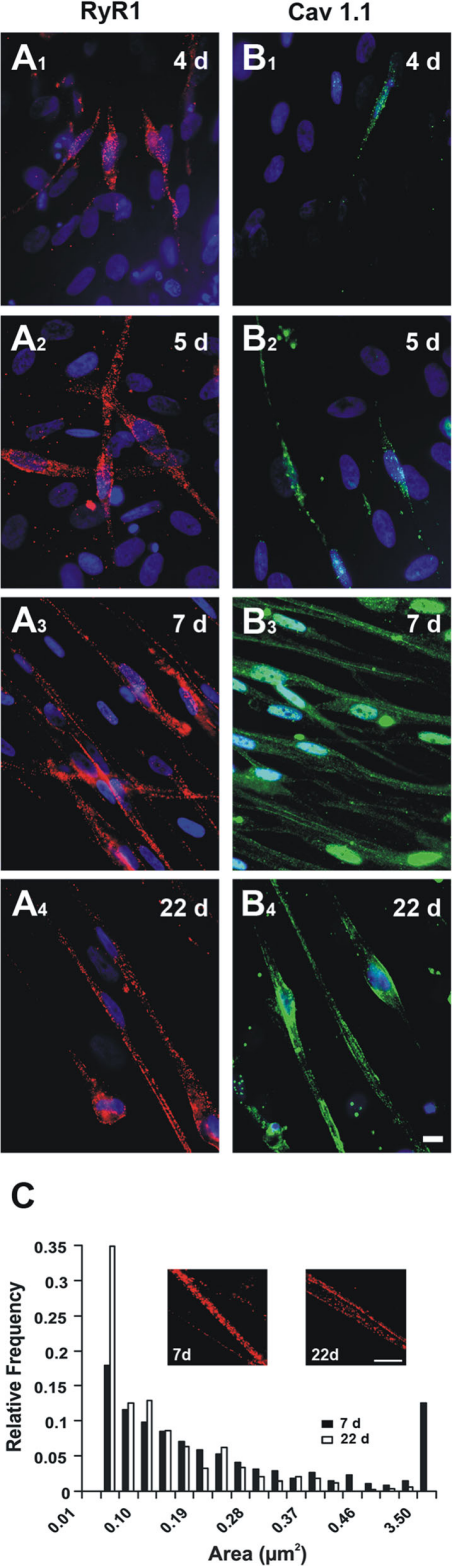
Immunofluorescence detection of RyR1 and Cav1.1 channels in differentiating human iPSC-derived skeletal myocytes. **A1**–**A4** Immunofluorescence labeling of RyR1 (red) and nuclei (blue) after 4 to 22 days of cell differentiation as indicated. **B1**–**B4** Immunofluorescence labeling of Cav1.1 (green) and nuclei (blue); same time sequence after differentiation as in **A1**–**A4**, scale: 10 μm. **C** Frequency distribution histograms of RyR1 immunofluorescent particle area at early (7 days) or mature (22 days) stages of differentiation. Insets represent enlarged areas of RyRs immunolabeled cells to highlight the difference in size of the fluorescent particles between 7 and 22 days post-differentiation (scale: 10 µm). Data were obtained from three independent experiments, and immunofluorescent particles were counted from 13 and 8 cells in 7- and 22-day-old myocytes, respectively

**Fig. 4:**
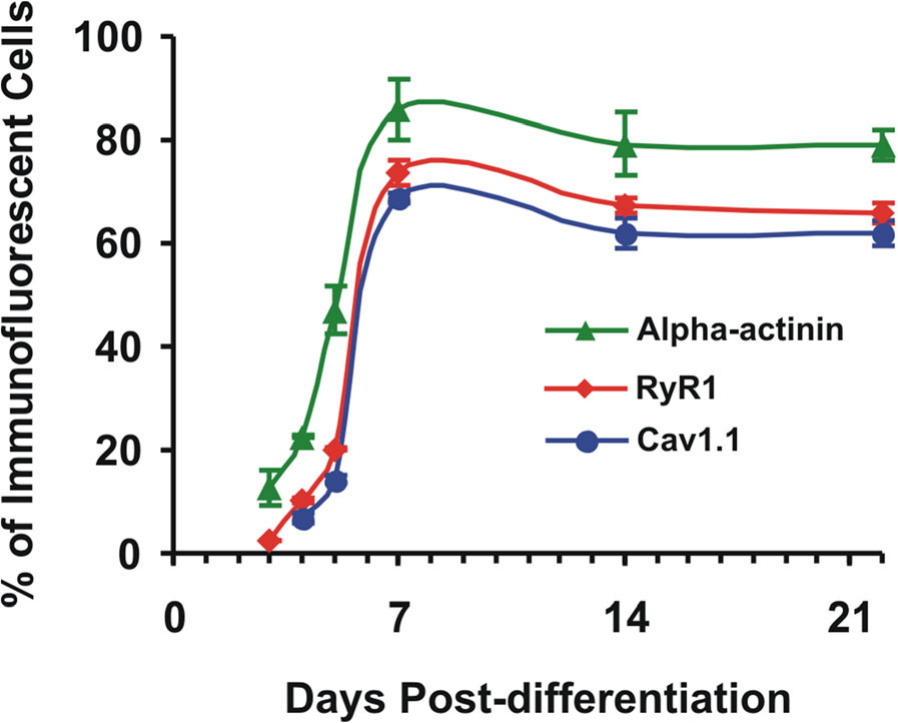
Percentage of α-actinin, RyR1, and Cav1.1 positive cells during differentiation. Symbols represent mean ± SEM of the percentage of α-actinin+/RyR1+/Cav1.1+ cells as counted from immunofluorescent microphotographs of iPSC-derived skeletal myocytes at different stages of their differentiation in culture. The total number of cells was determined by counting all Hoechst-stained nuclei on the microphotograph. Immunofluorescent cells were counted from four to ten frames per independent experiment (×60 magnification); three independent experiments were performed at each time point for each type of protein (labeled separately). Note that the percentage of immunolabeled cells for the three proteins reaches a stable level after the second week of differentiation

Optimal labeling of Cav1.1 and RyR1 channels was achieved by using antibodies from the same host. Consequently, double labeling could not be performed, and data are provided for each channel separately. Representative microphotographs of immunolabeled cells are shown in Fig. 3, and the percentage of RyR1- and Cav1.1-positive cells at different times of differentiation is depicted in Fig. 4.

RyR1 labeling was observed on the third day post-differentiation from elongated cells with flattened nuclei (Fig. 3A_1_, B_1_) and represented ~ 2.5% of the cells, but Cav1.1 labeling was negative (Fig. 4). This could indicate that RyR1 precedes Cav1.1 expression or that the latter is below the immunodetection threshold. On day 4, cells showing RyR1 labeling increased to 10%, and Cav1.1 labeling appeared in ~ 7% of the cells. On day 5, there was an increase in the proportion of RyR1 (~ 20%) and Cav1.1 (~ 14%)-labeled cells; however, the former still outnumbered the latter as can be seen from both Figs. 3A_2_, B_2_, and 4.

On day 7 post-differentiation, both RyR1 and Cav1.1 were noticeable in a large majority of cells. There was a drastic increase in the proportion of RyR1+ and Cav1.1+ cells (~ 74 and ~ 69%, respectively) as compared to day 5. Both channels seemed randomly distributed with no ordered pattern; RyR1 appeared as fluorescent patches that could be clearly delineated, while Cav1.1 labeling was less marked and more diffuse, consisting of hotspots of variable sizes contained in a more complex network of lower intensity. A relatively strong and diffuse labeling of Cav1.1 was noticed within the nucleus areas, for which we cannot provide at present a satisfactory explanation (Fig. 3A_3_, B_3_). The proportion of RyR1+ and Cav1.1+ cells declined slightly on day 14 (~  67 and 62%, respectively) then stabilized at this level at later stages (66 and 62%, respectively, on day 22 post-differentiation) as shown in Fig. 4 (see also Fig. 3A_4_, B_4_). Note that the developmental profile of these E-C coupling ion channels follows that of α-actinin, even though the percentage of actinin+ cells is slightly greater at all differentiation time points. Given the difference in antibodies sensitivity with a clear advantage to anti- α-actinin, one cannot draw conclusions from differences in absolutes values.

RyR1 channels appeared as discrete patches scattered throughout the cell, while Cav1.1 formed trails of fluorescent particles along the edges together with a more diffuse network across the cell. The spatial distribution of both channels did not follow the myofibril banding pattern. Overall, the organization in double rows of RyR1 and Cav1.1 typical of adult skeletal muscle, and which reflects the location of triads at the A-I junction, could be occasionally encountered but was not systematic in the present model. Nevertheless, for both channels, the size of the fluorescent patches seemed to decrease with cell maturation. To further investigate this change, we carried out a quantitative analysis of the fluorescent particles size. The accurate analysis was only possible for RyR1 channels since the fluorescence predominantly appeared in discrete particles that could be readily delineated and measured as opposed to Cav1.1 labeling which was both more discrete and diffuse, and hence difficult to quantify.

The frequency distribution of RyR1 immunoreactive particle sizes was compared between intermediate (7 days) and late (22 days) stages of differentiation, times at which a large number of cells expressing these channels were available for quantitative analysis. On day 7 post-differentiation, particle measurements revealed a wide-spread, asymmetrical distribution of the values (Fig. 3C). A large fraction of the particle size values ranged from 0.011 to 3.532 μm^2^, and 12.51% were above 0.519 μm^2^; the mean (± SEM) and median values were 0.263 ± 0.015 and 0.137 μm^2^, respectively (13 cells and a total of 671 immunoreactive particles)**.** At mature stages, the particle size distribution became narrower with values mainly ranging from 0.012 to 1.111 μm^2^, and only 2.12% of the total measurements exceeded 0.519 μm^2^. There was also a decrease in the mean (0.117 ± 0.006) and median (0.071 μm^2^) values with cell maturation (8 cells and a total of 501 immunoreactive particles). The difference in the mean values of RyR1 immunoreactive particle size between early and late stages was statistically highly significant (0.263 ± 0.015 and 0.117 ± 0.006 μm^2^, respectively, *p* < 0.001). Such changes with cell maturation may be explained by a decrease in the clustering of elementary fluorescent particles.

### Development of skeletal muscle-specific intracellular Ca^2+^ transient

**Fig. 5:**
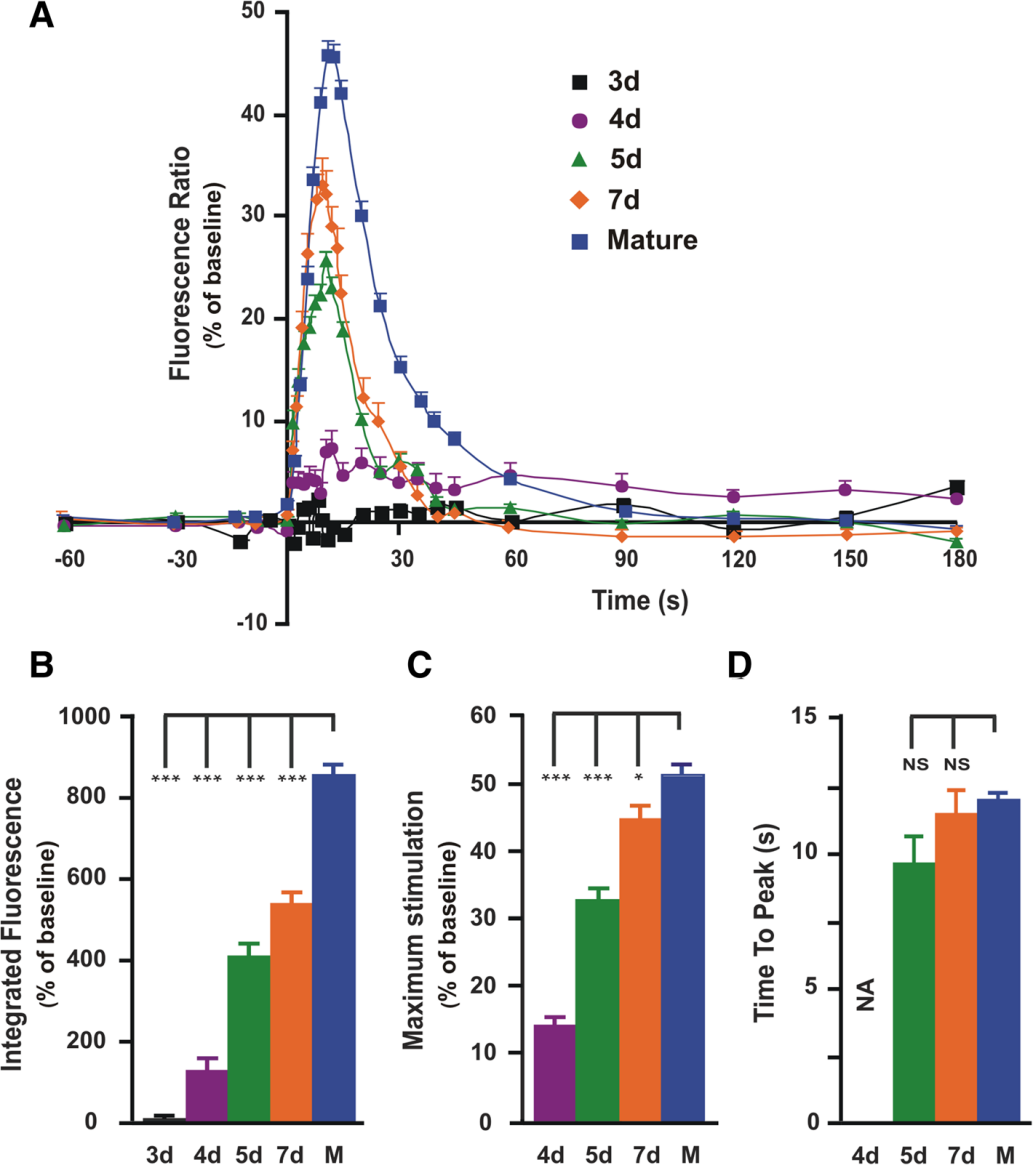
Depolarization-induced Ca^2+^ transients in differentiating human iPSC-derived skeletal myocytes. **A** Fura-2 ratiometric (340/380) fluorescence reflecting changes in intracellular Ca^2+^ level during 180 s of membrane depolarization by 50 mM extracellular [K^+^]. Symbols represent mean data (± SEM) obtained at different times post-differentiation as indicated (M: mature stages > 3 weeks post-differentiation). **B**–**D** Mean values (± SEM) of the three different characteristics of the Ca^2+^ signal at different times post-differentiation. Note that the value of time to peak was not included for day 4 (NA) as the signal has not reached a single peak profile necessary for this measurement

In a previous report [12], we showed that depolarization-induced intracellular Ca^2+^ signaling was absent in iPSC-derived muscle progenitors and occurred in the form of a typical Ca^2+^ transient after they have been differentiated in culture for 2–8 weeks. This event was considered among the decisive criteria for the physiological validation of the iPSC-based skeletal muscle model. In the present study, we looked into the precise timescale for the occurrence and maturation of this process. Changes in intracellular Ca^2+^ levels induced by membrane depolarization with 50 mM extracellular [K^+^] were examined at short post-differentiation intervals (3, 4, 5, and 7 days) during the first week when changes occur rapidly, then from 3 to 8 weeks, when the muscle cells have largely reached steady maturation. The development of intracellular Ca^2+^ signals induced by membrane depolarization with high external [K^+^], together with their main characteristics is depicted in Fig. 5. It also provides a statistical assessment of the degree of differentiation of each of the characteristics by comparison with the mature stage. As can be seen from Fig. 5A, there was no perceptible Ca^2+^ signaling on day 3 post-differentiation, followed on day 4 by a shallow, unsynchronized but consistent increase in intracellular Ca^2+^ level in elongated cells. Day 5 was a critical time point marked by the occurrence of a typical Ca^2+^ transient with a sharp upstroke and slow decay. From this stage onward, Ca^2+^ transients were present, and their intensity and kinetics changed with time until stabilization at mature stages. Changes over time of three different characteristics of the Ca^2+^ transient, i.e., the integrated fluorescence over the first 30 s of membrane depolarization, the maximum stimulation and the signal time to peak (see “Methods” section) are depicted in the histograms of Fig. 5B–D; mean values and statistical significance of differences observed between early and mature stages are given in Table 1.

**Table 1:** Changes of the intracellular Ca^2+^ signal characteristics with cell differentiation

Days	*n* (*N*)	Integrated fluorescence (% of baseline)	Peak (% baseline)	Time to peak (s)	Single decay, *τ* (s)	Double decay (s)		Contraction (% of Cells)
						*τ*_fast_	*τ*_slow_	
3	24 (3)	9.1 ± 8.0 ***	NA	NA	NA	NA	NA	0
4	28 (4)	129.7 ± 26.6 ***	13.9 ± 1.4 ***	NA	NA	NA	NA	0
5	24 (3)	411.7 ± 28.7 ***	32.6 ± 1.7 ***	9.6 ± 1.0	9.7 ± 1.1	NA	NA	0
7	42 (6)	539.6 ± 27.6 ***	44.6 ± 2.0 *	11.5 ± 0.9	8.4 ± 0.7	NA	NA	~ 25
Mature	107 (7)	857.2 ± 23.2	51.3 ± 1.3	12.2 ± 0.3	NA	9.1 ± 0.5	53.4 ± 3.6	~ 100

Statistical significance is indicated below the mean values: ***p < 0.001; **p < 0.01, *p < 0.5

*NA* not applicable, *n* total number of cells, *N* number of independent experiments performed

All numbers represent mean values. Results obtained during the first week of human iPSC-derived myogenic progenitors differentiation are compared with those of mature skeletal myocytes

On day 3, integrated fluorescence had a mean value of 9.1 ± 8.0 (fluorescence ratio × time (s) relative to the baseline), which is insignificant and most likely due to small fluctuations of the baseline. On day 4, there was a slight increase in integrated fluorescence from the baseline confirming the occurrence of an early form of Ca^2+^ signaling. Between days 4 and 5, integrated fluorescence increased sharply, followed by a moderate rise through the end of the first week of differentiation. During the maturation period, there was a further increase in integrated fluorescence reaching a mean value of 857.2 ± 23.2 at late stages (Fig. 5B, Table 1). In contrast to early stages, the increase in integrated fluorescence observed during the maturation period was mainly accounted for by a slowing of the decay phase of the Ca^2+^ signal rather than by an increase in the peak. This was corroborated by kinetic analysis of the decay phase showing that in a large fraction of 5- and 7-day-old skeletal myocytes (73.9 and 81.4%, respectively), Ca^2+^ decay followed a single exponential function with a comparable time constant (9.7 ± 1.1 and 8.4 ± 0.7 s, respectively). In contrast, at late stages, the best fitting required two exponential functions in 70% of the cells: a fast component similar to that found at early stages (*τ*_fast_ = 9.1 ± 0.5) and a slow component that appeared with cell maturation (*τ*_slow_ = 53.4 ± 3.6 s) (see Table 1).

The relative peak to the baseline or maximum stimulation increased rapidly between days 4 and 5 and then more gradually to the end of the first week. This characteristic did not change significantly during the maturation period (Fig. 5C and Table 1).

Time to peak could only be accurately measured in Ca^2+^ transients with a single peak, and hence was not applicable to unsynchronized Ca^2+^ signals recorded on day 4. Time to peak did not change significantly with time, and mean values obtained at different stages of development were all comparable (Fig. 5D and Table 1).

Depolarization-induced Ca^2+^ signals became effective in inducing myocyte contraction from day 7 post-differentiation. However, at this early stage, only 25% of the cells show mild contractions as compared to 100% of mature skeletal myocytes. The latter is illustrated in Additional file 4: Video S1, in which strong contractions are observed when the extracellular medium is switched to a depolarizing solution containing 50 mM K^+^ (no Ca^2+^ ions). It is worth noting that in a normal culture medium, the first spontaneous contractions can be noticed between 7 and 10 days post-differentiation and last throughout the maturation period (see Additional file 5: Video S2).

## Discussion

In a previous study [12], we showed that human iPax7-iPSC-derived muscle progenitors acquire, within the two first weeks of their differentiation in vitro, typical skeletal muscle structure and function. The excitation-contraction coupling process, which represents a strong discriminative marker between the skeletal and cardiac muscle [2], was clearly identified through depolarization-induced Ca^2+^ transients in human iPSC-derived skeletal myocytes. However, the developmental profile of this process and its relation to myofibrillogenesis remained to be clarified. In the present study, we show that Ca^2+^ signaling appears as soon as the fourth day post-differentiation and evolves rapidly towards a typical Ca^2+^ transient within a few days. This early phase of fast development is followed by a slower maturation process that culminates after the third week of differentiation. Essential subcellular structures and molecular components of the E-C coupling machinery take place during the first week of differentiation and interact to produce typical Ca^2+^ transients before the full development of sarcomeres. SR-TT junctions are clearly identified by electron microscopy as early as 4 days post-differentiation, and this coincides with the detection of muscle-specific RyRs (RyR1) and L-Type Ca^2+^ channels (Cav1.1) by fluorescence immunolabeling. However, at this early stage, the junctional space between SR and T-tubule membranes is not clearly delineated, and RyRs feet are hard to identify in the cleft. This observation is reminiscent of the first event in the developmental sequence of SR-TT junctions in mouse skeletal muscle [5]. On the functional side, Ca^2+^ signaling is present but its small size and slow kinetics both suggest an immature coupling process leading to the release of small amounts of Ca^2+^ ions in a desynchronized fashion. In the adult skeletal muscle, SR-TT junctions are highly ordered; RyR1 and Cav1.1 are organized into arrays composed of four Cav1.1 which associate with every other homotetrameric RyR1, forming alternate tetrads [23]. In addition, there is a bidirectional talk between RyR1 and Cav1.1 channels which depends on their precise arrangement and molecular interaction [8, 24, 25]. One could therefore speculate that at this early stage of SR-TT formation, most RyR1 and Cav1.1 channels are not aligned yet, and this lack of spatial organization could account for the minor Ca^2+^ signaling. Nonetheless, molecular rearrangement might rapidly occur at the SR-TT junctions as suggested by the tremendous increase in size and rate of Ca^2+^ release on day 5 and the occurrence of a Ca^2+^ transient reminiscent of that found in the skeletal muscle. The idea of a rapid rearrangement of RyR1 and Cav1.1 is supported by the clear appearance of RyRs feet and a well-delineated SR-TT cleft in electron micrographs at this time. Interestingly, the presence of this early Ca^2+^ transient was not accompanied by cell contraction. This could be due to immature contractile apparatus and/or to the insufficient amount of Ca^2+^ released into the cytosol. The first explanation is, in our view, the most plausible and is supported by studies showing that Z-bodies or nascent Z-bands, similar to those observed on day 5 in our system, do not support muscle contraction [20]. At this early stage of myofibrillogenesis, thin and thick filaments are aligned but intermingled with no A-I banding and are presumably unable to support the sliding process underlying the muscle contraction [26]. Furthermore, by the end of the first week following cell differentiation, when the A/I banding pattern becomes more manifest, depolarization-induced Ca^2+^ signals produce muscle contractions, and spontaneous contractions are also present under normal culture conditions.

After the first week of differentiation, the maturation process of the iPSC-derived muscle cells is characterized by further organization of the myofilaments as well as changes in SR-TT junctions, but these two structures do not ultimately reach the same degree of development. Indeed, by the end of the second week of differentiation, many myofibrils show a full banding pattern including the H-band and M-line. Thus, their contractile apparatus has reached a degree of organization that should sustain contraction in the most efficient manner. In parallel, SR cisternae and T-tubules become smaller, and the junctions seem better delineated and more central, i.e., closer to the myofibrils. Such changes are paralleled by a reduction in the size of RyR1 immunolabeling particles that plausibly reflects the spatial restriction of the SR cisternae in which they are expressed. Nevertheless, the occurrence of typical triads as well as their position at the A-I junction remains seldom in the present model. SR and T-tubule membranes seem to meet and form junctions at more or less random locations, and although more centrally localized at mature stages, SR-TT junctions remain irregularly distributed even after the myofibrils have reached their full organization. Thus, the orderly sarcomeric distribution of the SR-TT junctions typical of adult skeletal muscle [27] is not encountered in the present model. However, this does not prevent functional maturation of E-C coupling which is indicated by an increase in the Ca^2+^ transient peak and by the occurrence of a slow component in the decay phase. The former change can be explained by an increased expression of RyR1 and Cav1.1 channels, while the latter may be related to maturation of the Ca^2+^ buffering process that follows E-C coupling [28, 29]. Thus, by becoming larger and wider with cell maturation, depolarization-induced Ca^2+^ signals would generate a rise in intracellular Ca^2+^ that peaks and lasts long enough to enable strong myocyte contraction.

According to a number of developmental studies, the spatial positioning of SR-TT junctions is a slow and final process that occurs about 2 weeks after birth in rodents [5, 30–33]. In humans, change in orientation of the triads is still incomplete at 28 weeks of fetal development [34]*.*Interestingly, a similar timeframe is shared by muscle innervation which reaches its mature mono-synaptic form about 2 weeks after birth in rodents [35, 36] and within the first year of life in humans [37]. A possible link between innervation and the organization of the EC coupling apparatus is supported by in vitro studies showing that the cross-striated distribution of SR-TT junctions takes place only when the primary human skeletal muscle cells are innervated by co-cultured rat spinal neurons [38].

## Conclusion

SR-TT junctions occur in iPSC-derived skeletal muscle cells soon after the switch to differentiation conditions in vitro, and, although structurally immature, they are able to generate depolarization-induced intracellular Ca^2+^ transients typical of skeletal muscle. Such signals become effective in triggering muscle contraction as soon as the A-I banding pattern is in place. At later stages of differentiation, myofibrils are fully organized while SR-TT junctions are not, suggesting that their respective maturation is regulated by different signals. The final organization of the E-C coupling apparatus may depend on factors/conditions that are not provided or reproduced under these in vitro conditions, among which motor innervation represents an appealing candidate.

## Additional files

Additional file 1: Figure S1. Bright field photomicrographs of human iPSC-derived skeletal myocytes differentiating in culture. **A** Proliferating myogenic progenitors. **B**–**F** Cells photographed at different times (from 3 to 22 days; d = days) following the switch to differentiation conditions, (scale:100 μm). Notice the rapid elongation of the cells within the first week post-differentiation and the high density of mature skeletal myocytes on day 22. (TIFF 3627 kb)

Additional file 2: Figure S2. Alpha-actinin immunofluorescence and ultrastructure of human iPSC-derived skeletal myocytes on day 3 post-differentiation in culture. **A** Immunofluorescence labeling of α**-**actinin in one of the few elongated cells at this very early stage of differentiation. Note the punctuate labeling along the periphery of the cell. **B**_**1–3**_ Electron micrographs shown at increasing magnification to disclose structural details of the early cytoskeleton remodeling. The area occupied by nascent myofilaments is relatively limited and mainly localized at the periphery of the cell. Note the presence of many undifferentiated cells surrounding this early myocyte. (TIFF 3844 kb)

Additional file 3: Figure S3. Developing myofibrils in a 5-day-old human iPSC-derived skeletal myocyte. Nascent sarcomeres found at the center of the cell appear relatively more organized than those located at the periphery. Electron microphotographs were taken at low (upper image) and high (lower image) magnification from two different skeletal myocytes, 5 days after the switch to differentiation conditions. (TIFF 7644 kb)

Additional file 4: Video S1. Depolarization-induced contraction in mature human iPSC-derived skeletal myocytes. Membrane depolarization was triggered by 50 mM K^+^ in an extracellular medium devoid of Ca^2+^ ions. A typical Fura2 image sequence is first presented as 340/380 fluorescence ratio to reflect the increase in intracellular Ca^2+^ (from blue to red color) then as raw images captured using 380 nm excitation wavelength from which myocyte contractions can be more readily followed. To obtain a video file, Fura2 images (obtained in a property locked format, Aquacoscomos, Hamamatsu) were first converted into universal Tiff format using a screen capture software (Gadwin Systems Ltd). Tiff images sequence was then converted into a video file (.avi) with ImageJ software (NIH). (AVI 38635 kb)

Additional file 5: Video S2. Spontaneous contractions of 10-day-old human iPSC-derived skeletal myocytes. Live cell imaging on day 10 post-differentiation using time-sequence microphotography (61 frames at 100 ms interval converted to mp4 video format). Images were acquired with ×20 lens objective on a phase contrast TE2000 Nikon inverted microscope. Cells were bathed in standard culture medium, and images were captured at room temperature (~ 21 °C). (MP4 1740 kb)

## Abbreviations

Cav1.1: Voltage-gated Ca^2+^ channel (skeletal muscle type)
DHPR: Dihydropyridine receptor
E-C: Excitation-contraction
iPSC: Induced pluripotent stem cells
RyR1: Ryanodine receptor (skeletal muscle type)
SR: Sarcoplasmic reticulum
T-T: Transverse tubule

## References

[1] Schneider MF (1994). Control of calcium release in functioning skeletal muscle fibers. Annu Rev Physiol.

[2] Lamb GD (2000). Excitation-contraction coupling in skeletal muscle: comparisons with cardiac muscle. Clin Exp Pharmacol Physiol.

[3] Dulhunty AF (2006). Excitation-contraction coupling from the 1950s into the new millennium. Clin Exp Pharmacol Physiol.

[4] Flucher BE, Franzini-Armstrong C (1996). Formation of junctions involved in excitation-contraction coupling in skeletal and cardiac muscle. Proc Natl Acad Sci U S A.

[5] Takekura H, Flucher BE, Franzini-Armstrong C (2001). Sequential docking, molecular differentiation, and positioning of T-tubule/SR junctions in developing mouse skeletal muscle. Dev Biol.

[6] Avila G, Dirksen RT (2000). Functional impact of the ryanodine receptor on the skeletal muscle L-type Ca(2+) channel. J Gen Physiol.

[7] Paolini C, Fessenden JD, Pessah IN, Franzini-Armstrong C (2004). Evidence for conformational coupling between two calcium channels. Proc Natl Acad Sci USA.

[8] Nakai J, Dirksen RT, Nguyen HT, Pessah IN, Beam KG, Allen PD (1996). Enhanced dihydropyridine receptor channel activity in the presence of ryanodine receptor. Nature.

[9] Rios E, Brum G (1987). Involvement of dihydropyridine receptors in excitation-contraction coupling in skeletal muscle. Nature.

[10] Tanabe T, Beam KG, Powell JA, Numa S (1988). Restoration of excitation-contraction coupling and slow calcium current in dysgenic muscle by dihydropyridine receptor complementary DNA. Nature.

[11] Rios E, Pizarro G (1991). Voltage sensor of excitation-contraction coupling in skeletal muscle. Physiol Rev.

[12] Skoglund G, Laine J, Darabi R, Fournier E, Perlingeiro R, Tabti N (2014). Physiological and ultrastructural features of human induced pluripotent and embryonic stem cell-derived skeletal myocytes in vitro. Proc Natl Acad Sci USA.

[13] Bers DM (2002). Cardiac excitation-contraction coupling. Nature.

[14] Park IH, Arora N, Huo H, Maherali N, Ahfeldt T, Shimamura A (2008). Disease-specific induced pluripotent stem cells. Cell.

[15] Darabi R, Arpke RW, Irion S, Dimos JT, Grskovic M, Kyba M (2012). Human ES- and iPS-derived myogenic progenitors restore DYSTROPHIN and improve contractility upon transplantation in dystrophic mice. Cell Stem Cell.

[16] Darabi R, Perlingeiro RC (2016). Derivation of skeletal myogenic precursors from human pluripotent stem cells using conditional expression of PAX7. Methods Mol Biol.

[17] Chal J, Oginuma M, Al Tanoury Z, Gobert B, Sumara O, Hick A (2015). Differentiation of pluripotent stem cells to muscle fiber to model Duchenne muscular dystrophy. Nat Biotechnol.

[18] Grynkiewicz G, Poenie M, Tsien RY (1985). A new generation of Ca2+ indicators with greatly improved fluorescence properties. J Biol Chem.

[19] McKenna NM, Johnson CS, Wang YL (1986). Formation and alignment of Z lines in living chick myotubes microinjected with rhodamine-labeled alpha-actinin. J Cell Biol.

[20] Sanger JM, Mittal B, Pochapin MB, Sanger JW (1986). Myofibrillogenesis in living cells microinjected with fluorescently labeled alpha-actinin. J Cell Biol.

[21] Fry CS, Kirby TJ, Kosmac K, McCarthy JJ, Peterson CA (2017). Myogenic progenitor cells control extracellular matrix production by fibroblasts during skeletal muscle hypertrophy. Cell Stem Cell.

[22] Burkholder TJ, Lieber RL (2001). Sarcomere length operating range of vertebrate muscles during movement. J Exp Biol.

[23] Paolini C, Protasi F, Franzini-Armstrong C (2004). The relative position of RyR feet and DHPR tetrads in skeletal muscle. J Mol Biol.

[24] Protasi F, Paolini C, Nakai J, Beam KG, Franzini-Armstrong C, Allen PD (2002). Multiple regions of RyR1 mediate functional and structural interactions with alpha(1S)-dihydropyridine receptors in skeletal muscle. Biophys J.

[25] Sheridan DC, Takekura H, Franzini-Armstrong C, Beam KG, Allen PD, Perez CF (2006). Bidirectional signaling between calcium channels of skeletal muscle requires multiple direct and indirect interactions. Proc Natl Acad Sci U S A.

[26] Huxley H, Hanson J (1954). Changes in the cross-striations of muscle during contraction and stretch and their structural interpretation. Nature.

[27] Porter KR, Palade GE (1957). Studies on the endoplasmic reticulum. III. Its form and distribution in striated muscle cells. J Biophys Biochem Cytol.

[28] Calderon JC, Bolanos P, Caputo C (2014). The excitation-contraction coupling mechanism in skeletal muscle. Biophys Rev.

[29] Gehlert S, Bloch W, Suhr F (2015). Ca2+-dependent regulations and signaling in skeletal muscle: from electro-mechanical coupling to adaptation. Int J Mol Sci.

[30] Flucher BE, Andrews SB, Daniels MP (1994). Molecular organization of transverse tubule/sarcoplasmic reticulum junctions during development of excitation-contraction coupling in skeletal muscle. Mol Biol Cell.

[31] Schiaffino S, Margreth A (1969). Coordinated development of the sarcoplasmic reticulum and T system during postnatal differentiation of rat skeletal muscle. J Cell Biol.

[32] Edge MB (1970). Development of apposed sarcoplasmic reticulum at the T system and sarcolemma and the change in orientation of triads in rat skeletal muscle. Dev Biol.

[33] Flucher BE, Phillips JL, Powell JA, Andrews SB, Daniels MP (1992). Coordinated development of myofibrils, sarcoplasmic reticulum and transverse tubules in normal and dysgenic mouse skeletal muscle, in vivo and in vitro. Dev Biol.

[34] Walker SM, Schrodt GR, Currier GJ, Turner EV (1975). Relationship of the sarcoplasmic reticulum to fibril and triadic junction development in skeletal muscle fibers of fetal monkeys and humans. J Morphol.

[35] Redfern PA (1970). Neuromuscular transmission in new-born rats. J Physiol.

[36] Brown MC, Jansen JK, Van Essen D (1976). Polyneuronal innervation of skeletal muscle in new-born rats and its elimination during maturation. J Physiol.

[37] Hesselmans LF, Jennekens FG, Van den Oord CJ, Veldman H, Vincent A (1993). Development of innervation of skeletal muscle fibers in man: relation to acetylcholine receptors. Anat Rec.

[38] Tanaka H, Furuya T, Kameda N, Kobayashi T, Mizusawa H (2000). Triad proteins and intracellular Ca2+ transients during development of human skeletal muscle cells in aneural and innervated cultures. J Muscle Res Cell Motil.

