# Supplementary figures and images for: Development of the excitation-contraction coupling machinery and its relation to myofibrillogenesis in human iPSC-derived skeletal myocytes
Source: Skelet Muscle. 2018 Jan 5;8:1. doi: 10.1186/s13395-017-0147-5 (PMC5756430; doi:10.1186/s13395-017-0147-5)

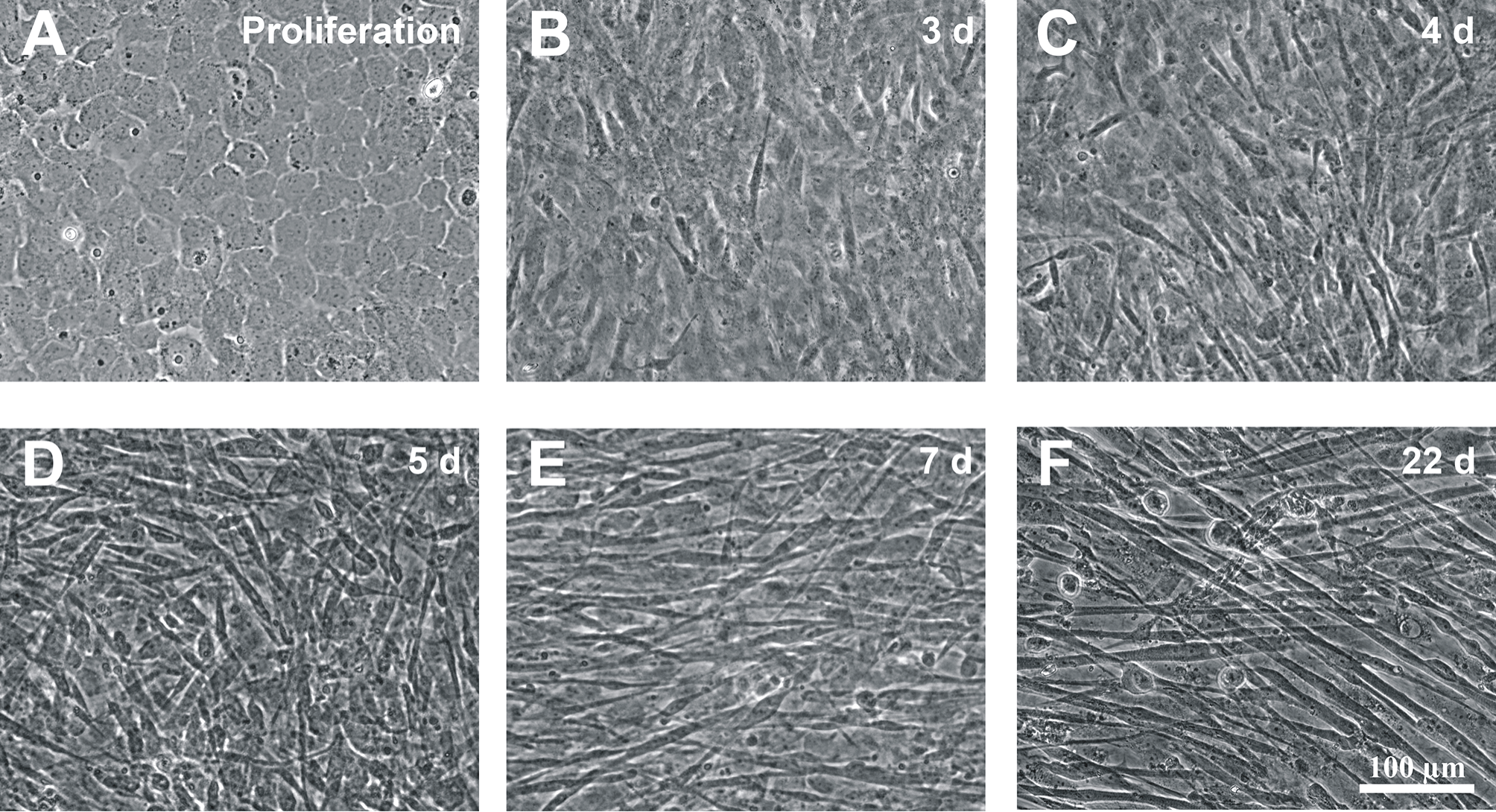

Supplement: Supplementary file 1 — Bright field photomicrographs of human iPSC-derived skeletal myocytes differentiating in culture. A Proliferating myogenic progenitors. B–F Cells photographed at different times (from 3 to 22 days; d = days) following the switch to differentiation conditions, (scale:100 μm). Notice the rapid elongation of the cells within the first week post-differentiation and the high density of mature skeletal myocytes on day 22. (TIFF 3627 kb) [file 13395_2017_147_MOESM1_ESM.tif]

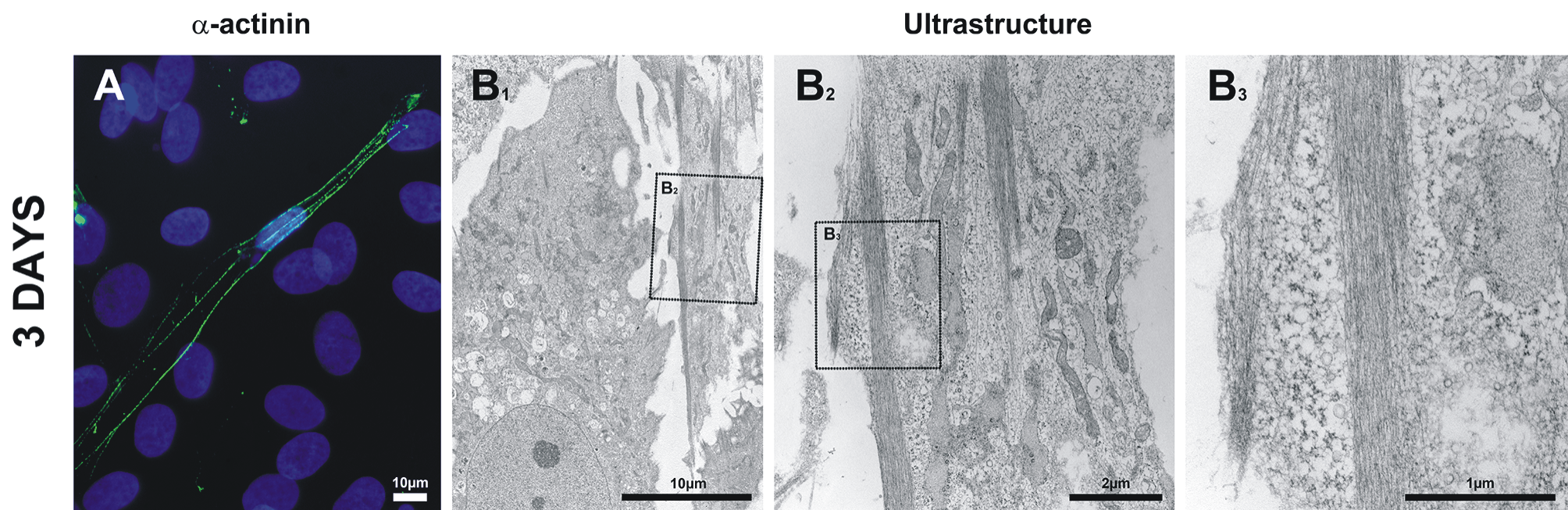

Supplement: Supplementary file 2 — Alpha-actinin immunofluorescence and ultrastructure of human iPSC-derived skeletal myocytes on day 3 post-differentiation in culture. A Immunofluorescence labeling of α-actinin in one of the few elongated cells at this very early stage of differentiation. Note the punctuate labeling along the periphery of the cell. B1–3 Electron micrographs shown at increasing magnification to disclose structural details of the early cytoskeleton remodeling. The area occupied by nascent myofilaments is relatively limited and mainly localized at the periphery of the cell. Note the presence of many undifferentiated cells surrounding this early myocyte. (TIFF 3844 kb) [file 13395_2017_147_MOESM2_ESM.tif]

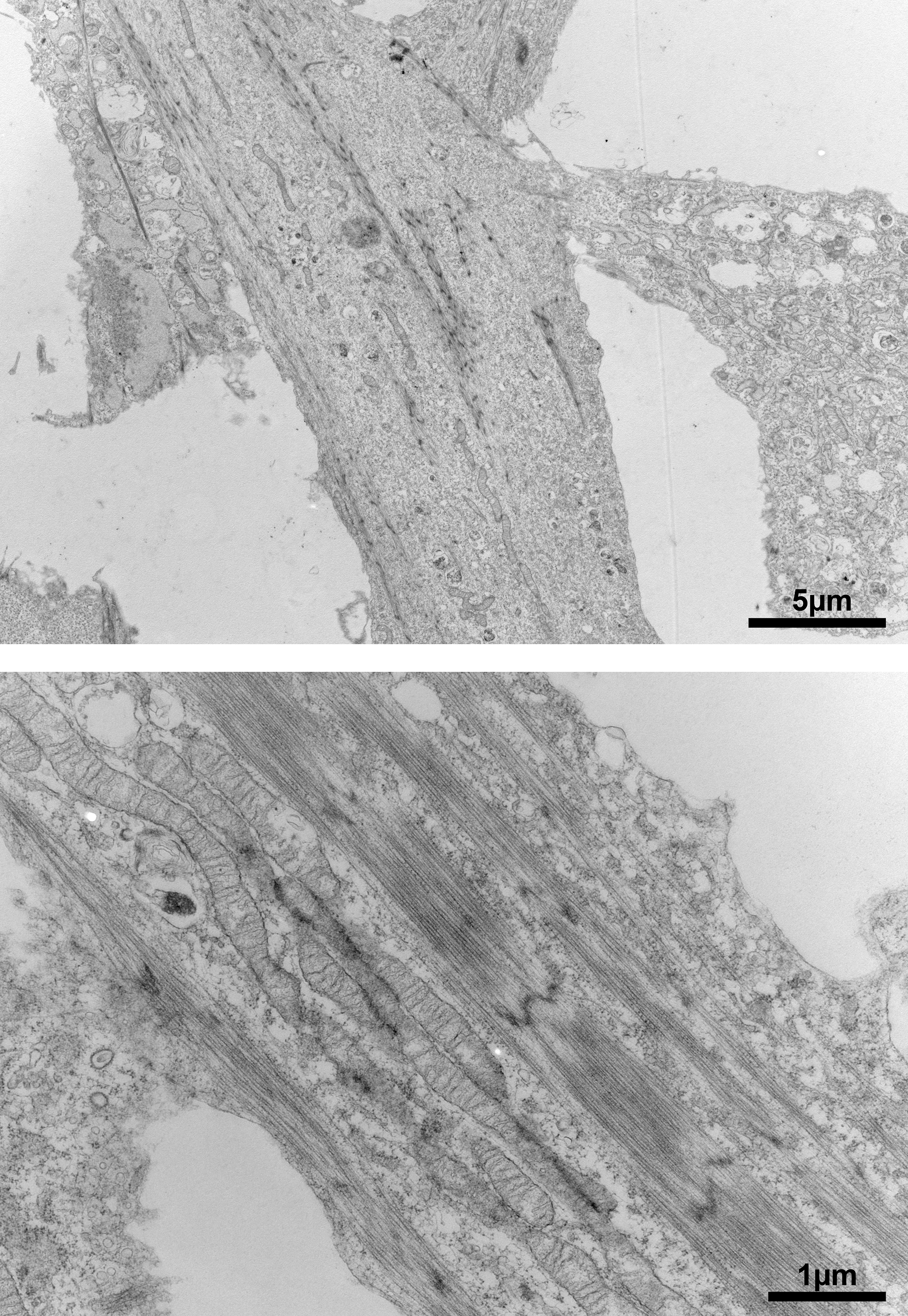

Supplement: Supplementary file 3 — Developing myofibrils in a 5-day-old human iPSC-derived skeletal myocyte. Nascent sarcomeres found at the center of the cell appear relatively more organized than those located at the periphery. Electron microphotographs were taken at low (upper image) and high (lower image) magnification from two different skeletal myocytes, 5 days after the switch to differentiation conditions. (TIFF 7644 kb) [file 13395_2017_147_MOESM3_ESM.tif]
